# SFESA: a web server for pairwise alignment refinement by secondary structure shifts

**DOI:** 10.1186/s12859-015-0711-0

**Published:** 2015-09-03

**Authors:** Jing Tong, Jimin Pei, Nick V. Grishin

**Affiliations:** Department of Biophysics and Department of Biochemistry, University of Texas Southwestern Medical Center at Dallas, 6001 Forest Park Road, Dallas, TX 75390-9050 USA; Howard Hughes Medical Institute, University of Texas Southwestern Medical Center at Dallas, 6001 Forest Park Road, Dallas, TX 75390-9050 USA

**Keywords:** Alignment refinement, Alignment improvement, Secondary structure evaluation

## Abstract

**Background:**

Protein sequence alignment is essential for a variety of tasks such as homology modeling and active site prediction. Alignment errors remain the main cause of low-quality structure models. A bioinformatics tool to refine alignments is needed to make protein alignments more accurate.

**Results:**

We developed the SFESA web server to refine pairwise protein sequence alignments. Compared to the previous version of SFESA, which required a set of 3D coordinates for a protein, the new server will search a sequence database for the closest homolog with an available 3D structure to be used as a template. For each alignment block defined by secondary structure elements in the template, SFESA evaluates alignment variants generated by local shifts and selects the best-scoring alignment variant. A scoring function that combines the sequence score of profile-profile comparison and the structure score of template-derived contact energy is used for evaluation of alignments. PROMALS pairwise alignments refined by SFESA are more accurate than those produced by current advanced alignment methods such as HHpred and CNFpred. In addition, SFESA also improves alignments generated by other software.

**Conclusions:**

SFESA is a web-based tool for alignment refinement, designed for researchers to compute, refine, and evaluate pairwise alignments with a combined sequence and structure scoring of alignment blocks. To our knowledge, the SFESA web server is the only tool that refines alignments by evaluating local shifts of secondary structure elements. The SFESA web server is available at http://prodata.swmed.edu/sfesa.

## Background

Homology modeling that constructs a structural model of a “query” protein based on its similarity to a homologous protein with known 3-dimensional structure (the “template”) remains the most reliable method of structure prediction. In most homology modeling methods, an essential step requires the input or construction of a pairwise sequence alignment between the query and the template, from which structurally equivalent residue pairs are deduced. Pairwise alignment is also the foundation for most multiple sequence alignment (MSA) methods. For example, the progressive method for MSA construction assembles a multiple sequence alignment by a series of pairwise alignments of sequences or pre-aligned groups [1].

Early methods of pairwise protein alignments apply dynamic programming algorithms that rely on general substitution matrices of amino acid residues and pre-defined gap penalties [2, 3]. Heuristic pairwise alignment tools such as BLAST [4] excel in speed and are suitable for sequence database searches. Numerical sequence profiles have been designed to incorporate information of homologous proteins to help aligning divergent sequences. PSI-BLAST [5] and HMMER [6] are examples of sequence-profile comparison methods that are generally more accurate than methods of sequence-sequence comparison. The subsequent development of profile-profile comparison methods [7–10] further enhanced alignment quality and the ability to detect homologous relationships. In addition to amino acid sequence profiles, predicted structural information, e.g., secondary structure and solvent accessibility, was also included in various alignment methods [11–13]. Three-dimensional structure information has been used in alignment construction methods in various ways, such as those based on structure-dependent profiles [14, 15] and a Monte Carlo-based alignment method that samples a set of moves of gapless alignment stretches and scores based on a template contact map [16].

Despite continuous method development in the alignment field, obtaining high-quality alignments for distantly related proteins remains a challenge. Alignment errors are still the main cause for the low quality of models built by homology. One common type of alignment error is the local misalignment, often by only a few residues, of secondary structure elements (α-helices and β-strands). Such errors often reflect the periodic nature of regular secondary structures. For example, many α-helices can be shifted by three or four residues while still maintaining a similar pattern of hydrophobic residues and polar residues. Therefore, one possible direction for refining an alignment lies in the generation of alignment variants by locally shifting secondary structure elements and evaluating the sequence and structure fitness of these alignment variants to determine which one is more likely to be correct.

Here we describe the SFESA web server, which refines pairwise protein alignments by evaluating alignment variants resulting from locally shifting secondary structure elements. The SFESA web server enables researchers to compute, refine, and evaluate pairwise alignments with a combined sequence and structure scoring of alignment blocks. The previous version of SFESA required the upload of a predefined template structure. In contrast, the new web server allows for a template to be specified by its PDB and chain identifiers. Furthermore, if no structure is provided, the SFESA server will search the database of sequences with experimentally determined 3D structures for the closest template, and this will then be used in the alignment refinement. The server facilitates further analysis of alignments at the level of secondary structure, providing detailed results of sequence and structure scores for local shifts of secondary structure elements. To our knowledge, the SFESA web server is the only online tool that refines alignments by evaluating local shifts of secondary structure elements.

## Implementation

### Overview of the SFESA alignment refinement method and procedure

Recently we developed SFESA [17], a method that refines pairwise protein sequence alignment by evaluating alignment variants generated from local shifts of secondary structure elements. SFESA first delineates alignment blocks from a starting pairwise protein alignment. Each alignment block corresponds to a regular secondary structural element (α-helix or β-strand as delineated by PALSSE [18]) in the template and the corresponding aligned region in the query. For each alignment block, SFESA generates a set of alignment variants by locally shifting query residues relative to template residues. Then, both a profile-based sequence score and a contact-based structure score of the aligned residue pairs in the original alignment block and the alignment variants are calculated. We have shown that the best-scoring alignment variant has the highest probability of being correct, e.g., showing the best agreement with the structure-based alignment.

SFESA uses two local shifting strategies to generate alignment variants with different treatments of gaps in the original alignment block. In the first strategy, up to 8 alignment variants are generated by shifting query residues up to four positions left or right relative to the template while maintaining the gap pattern in the original alignment block. However, we observed that gaps rarely occur in the middle of secondary structure elements in structure-based alignments. Therefore, in the second strategy, SFESA preprocesses the gap pattern in the original alignment block by eliminating gaps in the middle of the secondary structure elements. To achieve this, residues of an alignment block in both the query and template are shifted all the way to the left or right while all gaps are placed on the opposite side. Two preprocessed alignment blocks are generated: one by shifting residues to the left and filling the right side with gaps and the other by shifting residues to the right and filling the left side with gaps. Each of these two alignment variants is then used as a starting point to generate 8 additional alignment variants by ±4 shifts while keeping the modified gap patterns. This procedure gives rise to up to 18 (1 + 8 + 1 + 8) unique alignment variants (for details, see [17]).

For the sequence score, we use the profile-profile COMPASS score [7]. Sequence profiles are generated from PSI-BLAST multiple sequence alignments [5]. For the structure score, we define residue contacts based on the structure of the template. A residue contact is defined as a residue pair within a distance cutoff. In the template of an alignment, the residue contacts can be identified using the known structure of the template. We then evaluate the contact energy of corresponding contact residue pairs in the query that are inferred from query-template alignment. For example, if residue *i* in the template makes contact with residues *j*, *k*, and *m* in the template structure (i.e., contact pairs are (*i*, *j*), (*i*, *k*), and (*i*, *m*)), and the corresponding aligned residues for *i*, *j*, *k*, and *m* in the query are *i’* , *j’* , *k’* , and *m’* , respectively, then the inferred contact pairs in the query are (*i’* , *j’*), (*i’* , *k’*), and (*i’*, *m’*). The structure score for the aligned residue pair *i* and *i’* is CE(*i’* , *j’*) + CE(*i’* , *k’*) + CE(*i’* , *m’*), reflecting the structural fitness of the inferred query contact residue pairs. Here, CE is a matrix of the contact energy for residue pairs. We used two contact energy matrices: one is derived by Miyazawa and Jernigan [19] with contacts defined as residue pairs with side chain centers less than 6.5 Å, and the other is developed by us to best discriminate correct alignment variants from incorrect alignment variants (for details, see [17]). Regarding our derived contact matrix, the cutoff for contact definition is 6.5 Å between any side chain atoms of two residues.

In practice, the SFESA method uses a two-filter strategy to compare the scores of the original alignment block and the alignment variants and determines whether the original alignment block should be kept or changed to one of the alignment variants. The first filter checks if there are any alignment variants with a higher combined score I (S_comb_I_, a linear combination of sequence score and structure score) than the original alignment block. If none of the alignment variants has a S_comb_I_ higher than the original alignment block, SFESA rejects all the alignment variants and keeps the original alignment block. Otherwise, the alignment variant with the highest S_comb_I_ is selected and passed to the second filter. In the second filter, SFESA uses combined score II (S_comb_II_, a linear combination of sequence score and structure score) or an SVM score (S_SVM_) to compare the selected alignment variant and the original alignment block. If the selected alignment variant still has a higher S_comb_II_ or S_SVM_, SFESA will accept this alignment variant. Otherwise, SFESA keeps the original alignment block. The weights of the sequence score and structure score in S_comb_I_ and S_comb_II_ are optimized separately. S_SVM_ is a score reported by a support vector machine (SVM) that was trained to differentiate correct alignment variants from incorrect alignment variants by using a number of features including a COMPASS-based sequence score [7], a contact-based structure score, a solvent accessibility score and a secondary structure score (for details, see [17]). The solvent accessibility score is based on a three-by-three relative solvent accessibility substitution matrix derived from FAST [20] structural alignments of SCOP [21] domains. Similarly, the secondary structure score is based on a three-by-three secondary structure substitution matrix derived from FAST [20] structural alignments of SCOP [21] domains (for details, see [17]). The secondary structure is predicted by PSIPRED [22] for the query; the secondary structure information in DSSP [23] is used for the template. For each alignment block, starting from the N-terminus and proceeding to the C-terminus, SFESA decides whether to keep the original alignment block or to accept one of the alignment variants.

### The SFESA web server

**Fig. 1:**
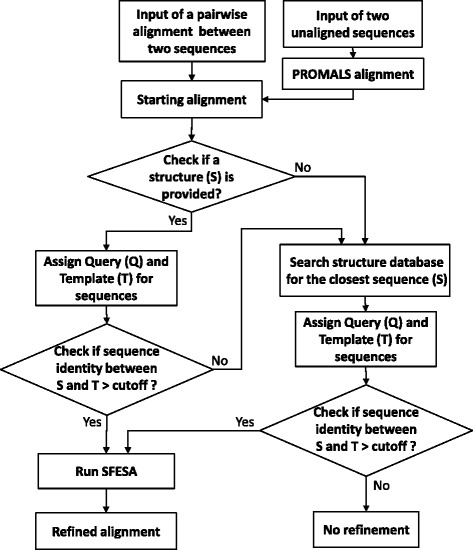
Flowchart of the SFESA web server. The sequence that is found to be the closest to the provided structure or the structure database is assigned as the Template (T). The other sequence is assigned as the Query (Q)

The SFESA web server is a tool for constructing, refining, and evaluating pairwise protein alignments (Fig. 1). The workflow of the server is shown in Fig. 1. Compared to the previously reported version of SFESA [17], in which a user must provide a structure for the template sequence, the updated server will search against our inhouse protein structure database to find the closest (to either sequence) homolog with available 3D structure to improve the alignment.

Users can input or upload sequences for the query and template either as a pairwise alignment or as two unaligned sequences in FASTA format. If two unaligned sequences are provided, the server uses PROMALS [11] to automatically construct a pairwise alignment. Input of a 3-dimensional structure (in pdb format) with high sequence similarity to the template is optional but recommended. A user can input a PDB identifier and a chain identifier, instead of a coordinate set, to directly use the structure from the RCSB PDB database [24]. If no structure is provided for the template, the server uses BLAST [5] to automatically search for homologs for either the query or template in a database of representative spatial structures and selects the best hit as the homologous structure, used as a template for structure score calculation.

Four SFESA alignment refinement modes are available in the web server: SFESA (O) uses up to 8 variants generated by ±4 shifts that keep the gap patterns of the original alignment block and the Miyazawa-Jernigan (MJ) [19] contact matrix for structure score calculation; SFESA (O + G) uses up to 18 variants by considering gap shifts and the MJ contact matrix; and SFESA (O + G + M) (default) uses a newly derived contact matrix in addition to gap processing; SFESA (O + G + M + S) differs from SFESA (O + G + M) in that an SVM-derived score is used in the second filtering step instead of S_comb_II_.

Several parameters are provided. One parameter is the sequence identity threshold between the template sequence and its homolog with a known 3D structure. SFESA refinement is applied only when the sequence identity between the template and its structure homolog is higher than the threshold (default = 0.5). Another parameter is the maximal number of residue positions to shift (default = 4, i.e., shifts are applied from −4 to +4 positions). Increasing this parameter generates more alignment variants, but also increases the probability that a wrong variant is accepted. The third parameter is the threshold for the fraction of non-gapped residue pairs above which an alignment block is used in the refinement process (default = 0.5). We also provide parameters for running and processing PSI-BLAST [5] results to generate the sequence profile used for the sequence score calculation, such as the number of iterations, the e-value inclusion cutoff, and a sequence identity cutoff to remove divergent hits.

**Fig. 2:**
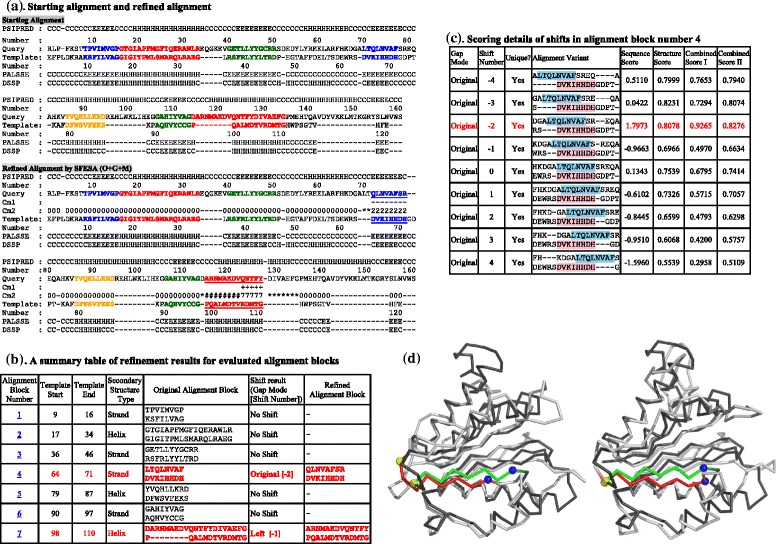
An example showing the output of the SFESA server and its ability to improve the alignment. (**a**) Output of the starting alignment and SFESA-refined alignment with secondary structure and colored alignment block. Predicted secondary structures for the query and the real secondary structures for the template are shown (“H”-Helix, “S”-Strand and “C”- Coil). “Number” shows the position number of the residue above the query and below the template, respectively. “Cm1” and “Cm2” represent the positional differences between the refined alignment and starting alignment. “Cm1” shows the sign of the query residue shifting (“ + ”: query residue shifted towards C-terminus; “-”: query residue shifted towards N-terminus) while “Cm2” shows the query residue shift number. If the query residue is aligned to a gap in both the starting and refined alignments, “Cm1” is left blank and “Cm2” shows the gap character “-”. If the query residue is aligned to one residue in the starting alignment but aligned to a gap in the refined alignment, “Cm1” is left blank and “Cm2” shows “*”. If a template residue is aligned to a gap in the starting alignment, both “Cm1” and “Cm2” are left blank. α-helix alignment blocks are shown alternately in red and orange. β-strand alignment blocks are shown alternately in blue and dark green. The refined alignment blocks are marked with underscores. (**b**) A table summarizing refinement results for the evaluated alignment blocks. The alignment block number is ordered from N-terminus to C-terminus. The sixth column indicates the refinement results of this alignment block. If refined, a format of “Gap mode [shift number]” is shown. Rows of the refined alignment blocks are colored red. (**c**) One example of the scoring details of shifts for alignment block number 4. This table contains the original alignment block and all alignment variants. The first column in the table is gap mode. There are three gap modes if there are gaps in this alignment block: Original (no change of the original alignment block), Left (residues in alignment blocks are aligned all the way to the left while all gaps are put to the opposite side before shifting) and Right (residues in alignment blocks are aligned all the way to the right while all gaps are put to the opposite side before shifting). The second column is the shift number. The third column indicates if such a variant is a unique one or the same as a variant shown previously. The fourth column shows the alignment variants with extended residues in both ends. The residues in the original alignment block are colored blue (query) and pink (template). The last four columns show the sequence score, structure score, combined score I and combined score II of each alignment variant. The row colored red corresponds to the alignment variant that is the final choice in the refined alignment. (**d**). Structure superpositions of query structure models (light grey ribbon) and query real structure (dark grey ribbon). Structure models were generated by MODELLER based on the starting alignment (left panel) and the SFESA-refined alignment (right panel). The strand (“QLNYAFSR”) in alignment block number 4 is highlighted. This strand is shown in red and green in the structure model and the real structure, respectively. Blue spheres and yellow spheres mark the N-terminal boundary (“Q”) and the C-terminal boundary (“R”), respectively

The output page of the SFESA web server includes the starting alignment (the input alignment or in the case of the input of unaligned sequences, the automatically generated PROMALS alignment), the refined alignment, and the refinement details for each evaluated alignment block. Figure 2a, b, and c shows one example of the output page. The first part of the output page (Fig. 2a) contains the starting alignment and the refined alignment with colored alignment blocks. PSIPRED [22] predicts secondary structure elements of the query and secondary structure elements of the template are based on PALSSE [18] and DSSP [23]. These predicted elements are shown above the query sequence and below the template sequence, respectively. Evaluated alignment blocks are depicted in red and orange for α-helices and blue and dark green for β-strands to distinguish them. In the SFESA-refined alignment, the modified alignment blocks are marked with underscores.

The second part of the output page (Fig. 2b) is a table summarizing the refinement results of the evaluated alignment blocks, numbered from the N-terminus to the C-terminus. Each row in the table provides the element start and end position numbers in the template, the element secondary structure type, the original alignment block, the shift result, and the refined alignment block. The shift result column shows Gap Mode and Shift Number. Gap Mode can be “Left” (gap pattern preprocessed by moving residues all the way to the left), “Right” (gap pattern preprocessed by moving residues all the way to the right), or “Original” (no gap preprocessing). Shift Number (in brackets) is the number of positions the residues in the query are shifted by, relative to the template. The “+” and “−” signs in Shift Number denote that the query residues in the alignment block are shifted towards the C-terminus or the N-terminus, respectively. If no alignment variant was accepted for an alignment block (i.e., the original refinement retained), “No shift” is shown in the shift result column and “-” is shown in the column of Refined Alignment Block. The third part of the output page contains tables with scoring details for the alignment variants. A table is provided for each alignment block evaluated by SFESA and presents each alignment variant and its sequence score, structure score, and combined scores I and II. Figure 2c provides an example for alignment block number 4. The residues in the original alignment block are colored blue and pink for the query and the template, respectively. The scoring details for alignment variants may help users manually evaluate and select alternative alignments.

## Results

### Test on the SABmark benchmark

We tested SFESA on the SABmark benchmark database of alignments [25]. The SABmark database has two alignment sets with different levels of sequence similarity and thus different levels of difficulty for alignment. The Twilight Zone set was created by selecting structurally similar domains at the SCOP [21] fold level and contains sequences with very low to low similarity. The Superfamilies set was created by selecting structurally similar domains at the SCOP superfamily level and contains sequences with low to intermediate similarity. We tested the ability of SFESA to refine alignments generated by several alignment methods, such as PROMALS [11], HHpred [12], and CNFpred [13]. Here HHpred was used in the global alignment mode because its local alignment mode often results in short alignments and shows lower alignment accuracy than global alignments.

We used the reference-dependent Q-score as the assessment. The Q-score is the fraction of correctly aligned residue pairs in a test alignment among all aligned residue pairs in a reference alignment. In this paper, the range of Q-score values is from 0 to 100 (e.g. 100 means 100 % agreement with reference).

SFESA can improve the PROMALS Q-score from 46 to 48 for the Twilight Zone set and from 71 to 72 for the Superfamilies set (Table 1). PROMALS-based SFESA outperforms other advanced alignment methods, such as HHpred and CNFpred. In practice, SFESA (O + G + M) and SFESA (O + G + M + S) produced similar results that are on average better than SFESA (O) and SFESA (O + G). Furthermore, SFESA also improves alignments generated by other methods (Table 1), including HHpred and CNFpred.

**Table 1:** Evaluation of alignment methods on the SABmark benchmark

Methods	SABmark_TWI (209)	SABmark_SUP (425)
PROMALS	46.2	71.10
SFESA (O) + PROMALS	47.3	71.30
SFESA (O + G) + PROMALS	48.0	71.80
SFESA (O + G + M) + PROMALS	47.9	71.90
SFESA (O + G + M + S) + PROMALS	**48.1**	**72.10**
HHpred	40.7	68.9
SFESA (O) + HHpred	40.6	69.0
SFESA (O + G) + HHpred	41.3	69.1
SFESA (O + G + M) + HHpred	**41.4**	**69.6**
SFESA (O + G + M + S) + HHpred	41.3	69.4
CNFpred	41.5	66.1
SFESA (O) + CNFpred	41.6	66.4
SFESA (O + G) + CNFpred	42.3	67.0
SFESA (O + G + M) + CNFpred	**42.4**	**67.4**
SFESA (O + G + M + S) + CNFpred	42.2	66.9

Average Q-scores of two SABmark [25] data sets (‘TWI’ for ‘Twilight Zone’ set, ‘SUP’ for ‘Superfamilies’ set) are shown. The Q-score is the number of correctly aligned residue pairs in the test alignment divided by the total number of aligned residue pairs in the reference alignment. One pair of domains is selected randomly from each group in the SABmark sets. For each set, the number in the parentheses is the number of alignments tested. Bold numbers indicate the best performance in the subsection

### An example of an alignment improved by the SFESA server

In the example shown in Fig. 2, the input consisted of two SCOP domains, d1ja1a3 (query) and d2piaa2 (template), and the 3D structure of the template. SFESA used PROMALS to obtain the starting alignment, which was refined to generate the refined alignment with the default option SFESA (O + G + M). Out of the seven alignment blocks evaluated by SFESA, five alignment blocks were kept without shifts and two alignment blocks were modified according to SFESA refinement scores (Fig. 2b). Both of these modified alignment blocks are in better agreement with the Dali structural alignment [26] of the query and the template compared to the original alignment blocks. We generated structure models for the query based on the starting alignment (Fig. 2d, left panel) and the refined alignment (Fig. 2d, right panel). Both models (in light grey and red ribbons) were superimposed upon the real structure of the query (in dark grey and green ribbons). The GDT-TS scores [27] for models generated from the starting alignment and the refined alignment are 57.7 and 67.1, respectively. The query secondary structure element in the fourth evaluated alignment block is highlighted in both structure superpositions (green for the real structure and red for the model). This element, misaligned by two residues in the starting alignment (Fig. 2d, left panel), has been corrected in the refined alignment (Fig. 2d, right panel). As a result, the RMSD for this secondary structure element between the model and the real structure improved from 5.3 Å for the model generated by the starting alignment to 2.0 Å for the model generated by the refined alignment.

## Discussion

Despite many significant research efforts, it is still challenging to correctly align weakly similar but homologous protein sequences. Alignment errors remain the main reason for the poor quality of homology-based models. Refining the alignments generated by automatic methods is a promising approach for increasing alignment quality. We found that secondary structure elements are often misaligned by only a few residues and that more accurate solutions can be identified within a limited set of local shifts of secondary structure elements. Therefore, we developed the SFESA method in order to refine alignments by evaluating the alignment variants generated by local shifts of template-defined secondary structures.

In the SFESA scoring system, both a profile-based sequence score and a novel contact-based structure score of the aligned residue pairs in the original alignment block and the alignment variants are calculated. Thus, an insufficient number of contacts can limit the quality of the alignment refinement. We found that structure scoring works well when there are sufficient contacts in the template as well as sufficient corresponding aligned residues in the query [17]. However, if a secondary structure element is involved in too few contacts (e.g. exposed edge β-strands), these contacts are insufficient to define a complete structural environment. SFESA is less effective in these cases. This observation suggests that dedicated efforts on misaligned blocks with insufficient contacts are required to improve alignments further.

## Conclusions

SFESA is a web-based tool to compute, refine, and evaluate pairwise alignments with a combined sequence and structure scoring of alignment blocks. Taking a pairwise alignment as input, the SFESA web server searches against an in-house database of protein spatial structures to find the closest homolog of either sequence. It then refines the pairwise alignment by combining the sequence profile similarity and residue-residue contact information that were obtained from the homolog with the structure. Finally, it facilitates further analysis of the alignment results at the level of secondary structure, providing details about scoring for all shifts of secondary structure elements.

## Availability and requirements

**Project name:** SFESA, a pairwise alignment refinement tool.

**Project home page:**http://prodata.swmed.edu/sfesa.

**Operating system(s):** Platform independent.

**Programming language:** Perl.

**Other requirements:** Internet connection, a browser.

**Any restrictions to use by non-academics:** None.
